# Identification of novel biomarkers in Hunner’s interstitial cystitis using the CIBERSORT, an algorithm based on machine learning

**DOI:** 10.1186/s12894-021-00875-8

**Published:** 2021-08-16

**Authors:** Kaining Lu, Shan Wei, Zhengyi Wang, Kerong Wu, Junhui Jiang, Zejun Yan, Yue Cheng

**Affiliations:** 1 Department of Urology, Ningbo First Hospital, 59, Liuting Street, Ningbo, 315010 Zhejiang People’s Republic of China; 2 Department of Urology and Nephrology, Ningbo First Hospital, Ningbo Hospital of Zhejiang University, 59, Liuting Street, Ningbo, 315010 Zhejiang People’s Republic of China; 3 Department of Respiratory and Critical Care Medicine, People’s Hospital Affiliated to Ningbo University, Yinzhou People’s Hospital, Ningbo, People’s Republic of China; 4 Department of Central Laboratory, People’s Hospital Affiliated to Ningbo University, Yinzhou People’s Hospital, Ningbo, People’s Republic of China

**Keywords:** Bioinformatic, Immune system diseases, Mast cells, Interstitial cystitis, Hunner-type interstitial cystitis

## Abstract

**Background:**

Hunner’s interstitial cystitis (HIC) is a complex disorder characterized by pelvic pain, disrupted urine storage, and Hunner lesions seen on cystoscopy. There are few effective diagnostic biomarkers. In the present study, we used the novel machine learning tool CIBERSORT to measure immune cell subset infiltration and potential novel diagnostic biomarkers for HIC.

**Methods:**

The GSE11783 and GSE57560 datasets were downloaded from the Gene Expression Omnibus for analysis. Ten HIC and six healthy samples from GSE11783 were analyzed using the CIBERSORT algorithm. Gene Set Enrichment Analysis (GSEA) was performed to identify biological processes that occur during HIC pathogenesis. Finally, expression levels of 11 T cell follicular helper cell (Tfh) markers were compared between three healthy individuals and four patients from GSE57560.

**Results:**

Six types of immune cells in HIC from GSE11783 showed significant differences, including resting mast cells, CD4^+^ memory-activated T cells (CD3^+^ CD4^+^ HLA-DR^+^ cells), M0 and M2 macrophages, Tfh cells, and activated natural killer cells. Except for plasma cells, there were no significant differences between Hunner’s lesion and non-Hunner’s lesion areas in HIC. The GSEA revealed significantly altered biological processes, including antigen–antibody reactions, autoimmune diseases, and infections of viruses, bacteria, and parasites. There were 11 Tfh cell markers with elevated expression in patients from GSE57560.

**Conclusion:**

This was the first demonstration of Tfh cells and CD3^+^ CD4^+^ HLA-DR^+^ cells with elevated expression in HIC. These cells might serve as novel diagnostic biomarkers.

**Supplementary Information:**

The online version contains supplementary material available at 10.1186/s12894-021-00875-8.

## Background

Interstitial cystitis (IC), also known as bladder pain syndrome (BPS), is a chronic condition characterized by painful lower urinary tract symptoms [1]. The condition affects millions of people and significantly impairs their quality of life. The diagnosis is challenging, and there are limited treatment options. Using cystoscopy, IC can be divided into two phenotypes: Interstitial cystitis with Hunner’s lesion (HIC) and Interstitial cystitis without Hunner lesions (NHIC) [1]. HIC occurs in between 10 and 20% of all IC patients [2]. Another report stated that the worldwide prevalence of HIC ranged from 3.5 to 56% [3]. HIC is distinguished from NHIC based on pathological and clinical differences [4]. Abnormal immunity is a well-known histological feature of HIC [5]. In the past, several gene expression studies credibly indicated dysfunctional inflammatory cytokines and autoimmune pathways HIC [6, 7]. The underlying etiology and mechanisms involved in phenotypes with HIC remain unclear.

The precise role of autoimmunity in HIC has not yet been clarified or compared to other autoimmune diseases [4]. To date, many hypotheses have been proposed, including infiltration and accumulation of mast cells, clonal expansion of infiltrating lymphocytes, and increased urothelial cell apoptosis [8, 9]. Studies showed that immune cells and autoantibodies are closely related to IC/HIC [10, 11].

Nevertheless, there are no diagnostic cell biomarkers of HIC, and understanding of immune cell subset infiltration in HIC is incomplete. Classic methods for studying immune cell subsets such as flow cytometry and immunohistochemistry rely on limited phenotypic markers. Tissue disaggregation before flow cytometry can lead to cell loss or damage, thereby distorting the results [12].

The CIBERSORT algorithm, developed by Newman et al., provides estimations of the abundances of member cell types in mixed cell populations using gene expression data. The approach is suitable for RNA mixtures from nearly any tissue [13]. A leukocyte gene signature matrix containing 547 genes was pre-installed with CIBERSORT to distinguish 22 human immune cell phenotypes, including monocytes, T cells, B cells, neutrophils, macrophages, plasma cells, natural killer cells, eosinophils, dendritic cells, mast cells, and several subsets of the above. The samples can be deconvolved by applying linear support vector regression (a novel machine learning approach) [13]. After permutation analysis, the relative fraction of 22 immune cell subsets can be assessed [13]. By exhibiting strong performance on its large scale and substantially improved accuracy, novel therapeutic targets and cellular biomarkers identification can be made possible. Nevertheless, this pioneering algorithm has not yet been applied to HIC.

Therefore, in the current study, gene expression data from ten HIC samples and six healthy samples stored in GSE11783 of the GEO database were analyzed. Novel predicted diagnostic biomarkers and the proportion of 22 immune cell subsets were assessed using CIBERSORT. GSEA assay was conducted to find significant enrichment of Kyoto Encyclopedia of Genes and Genomes (KEGG) pathways and Gene Ontology (GO) terms. Finally, we used samples from GSE57560 to validate our findings.

## Materials and methods

### Microarray data processing

Our data were derived from the public databases of GEO from NCBI. We used “interstitial cystitis” and “bladder pain syndrome” as keywords. The data selection criteria were as follows: (1) the study type was human tissue expression profiling by array; and (2) the samples had not been processed by other factors. Following these criteria, we retrieved three microarray datasets (GSE11783, GSE 57560, and GSE28242). However, the sample type of GSE28242 belongs to urine sediment. The immune cells may be unstable due to several factors; therefore, GSE28242 was finally excluded. GSE11783 contained ten HIC samples from five patients and six healthy donors based on the GPL570 platform. In this array, all five HIC patients had Hunner's lesion. Two bladder biopsies were analyzed for each HIC patient, one from Hunner’s lesion area and one from a non-lesion area. Six normal healthy controls were included with no cystoscopic findings.

We performed CIBERSORT algorithm analysis on GSE11783 in subsequent experiments. GSE57560 contained three normal samples, nine IC samples with normal bladder capacity, and four IC samples with low bladder capacity. Of these, two samples of four IC with low bladder were of the HIC type. However, details were not listed concerning the one-to-one correspondence between the disease subset of HIC/NHIC and the sample identifications. Finally, we selected four more disease characterized samples (IC with low bladder capacity) and three normal controls from GSE57560 as the validation group to detect T cell follicular helper cells (Tfh). The normalized expression matrix and sample information of GSE11783 and GSE57560 were downloaded from the GEO database.

### Immune infiltration analysis of GSE11783 using the CIBERSORT algorithm

To analyze significant differential expression of various immune cell types, we compared the HIC and control groups. Using the CIBERSORT algorithm, 22 subpopulations of immune cells in IC tissues were tested. These immune cells are as follows: memory B cells, naive B cells, naive CD4^+^ T cells, CD8^+^ T cells, activated memory CD4^+^ T cells, resting memory CD4^+^ T cells, Tfh, regulatory T cells, gamma-delta T cells, plasma cells, resting natural killer (NK) cells, activated NK cells, monocytes, M0 macrophages, M1 macrophages, M2 macrophages, resting mast cells, activated mast cells, resting dendritic cells, activated dendritic cells, eosinophils, and neutrophils. The abundance levels of 22 immune cells between the ten HIC tissues and the six normal controls were analyzed using the “vioplot package” in R version 3.6.1. The results were considered accurate with a cutoff standard *P*-value < 0.05. To further understand the relationship between these immune cells, Pearson correlation coefficients were used to calculate the correlations among these constituents. Principal component analysis (PCA) was performed to determine whether there were clear boundaries in immune cell infiltration between IC tissues and healthy controls. The immune cell infiltration characteristics were further compared between Hunner’s lesion area tissues and non-Hunner’s lesion area tissues in the IC group.

### Differentially expressed gene identification and Gene Set Enrichment Analysis of GSE11783

The fold-change of gene expression between the HIC group and control group was calculated, and the differentially expressed genes (DEGs) list was generated according to the change of |log2FC|. Gene Set Enrichment Analysis (GSEA) identifies functional enrichment in omics data by comparing genes in various gene sets [14]. A gene set is defined as a set of related genes that share relationships such as localization, pathways, functions, or other features. Compared to single-gene analysis, GSEA improves statistical power [14]. GSEA was conducted using clusterProfiler package in R. To gain mechanistic insight into enrichment results, GSEA-based enriched GO and KEGG analyses were performed [15–17].

### Tfh cell markers expression test in tissue samples from GSE57560

Eleven Tfh cell markers (CXCR5, PD-1, ICOS, SAP, CD200, IL-21, CXCR4, CXCL13, BTLA, SLAM, and CCR7) were obtained from the literature as detection indicators [18]. The expression levels of these cell markers were compared between normal tissues (n = 3) and IC tissues with low bladder capacity (n = 4) from GSE57560. Statistical analysis was conducted using GraphPad Prism.

## Results

### Main findings

**Fig. 1:**
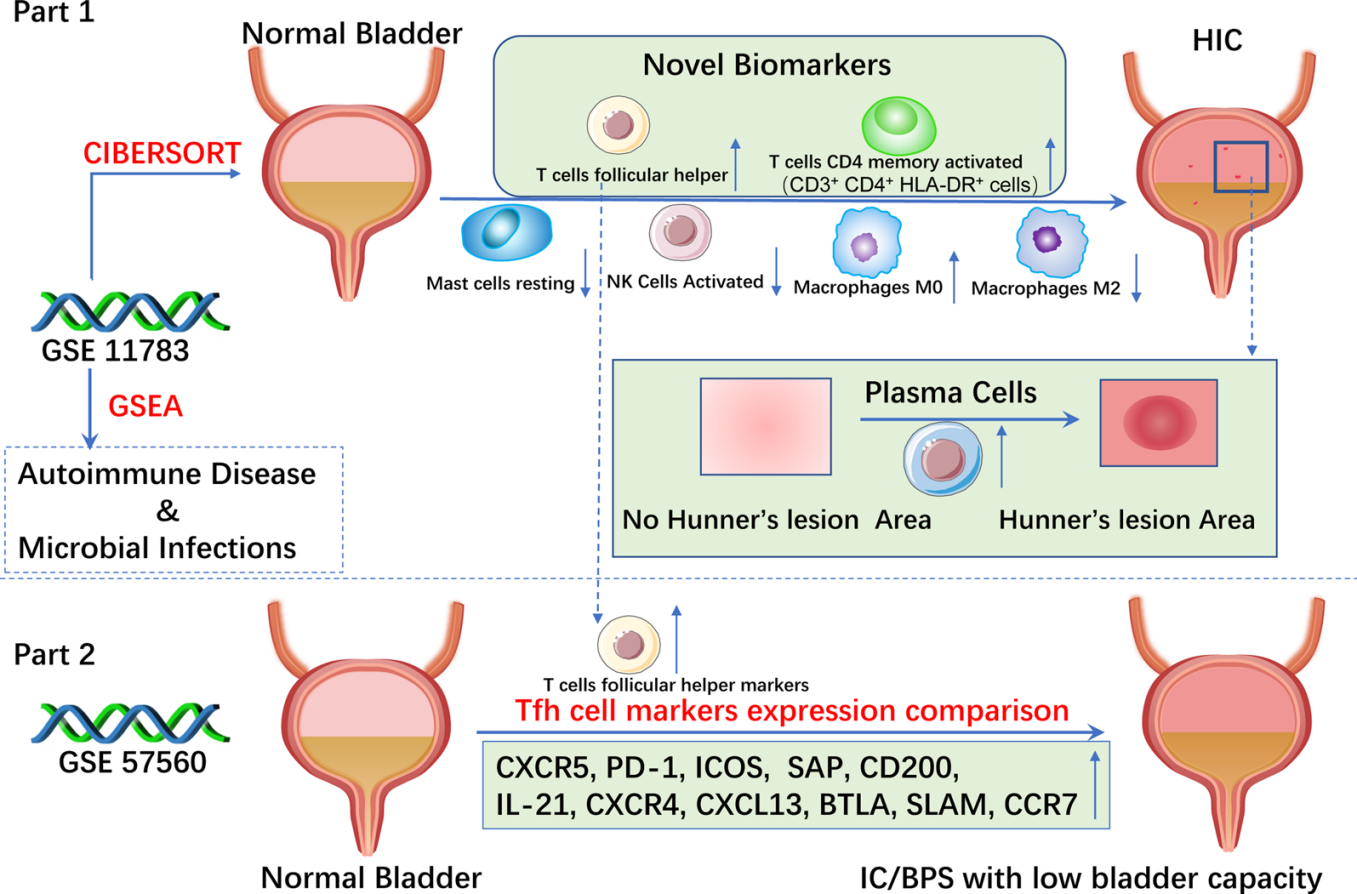
Major findings of this study

The main findings of the current work were displayed in Fig. 1.

### Immune cell infiltration characteristics of HIC

**Fig. 2:**
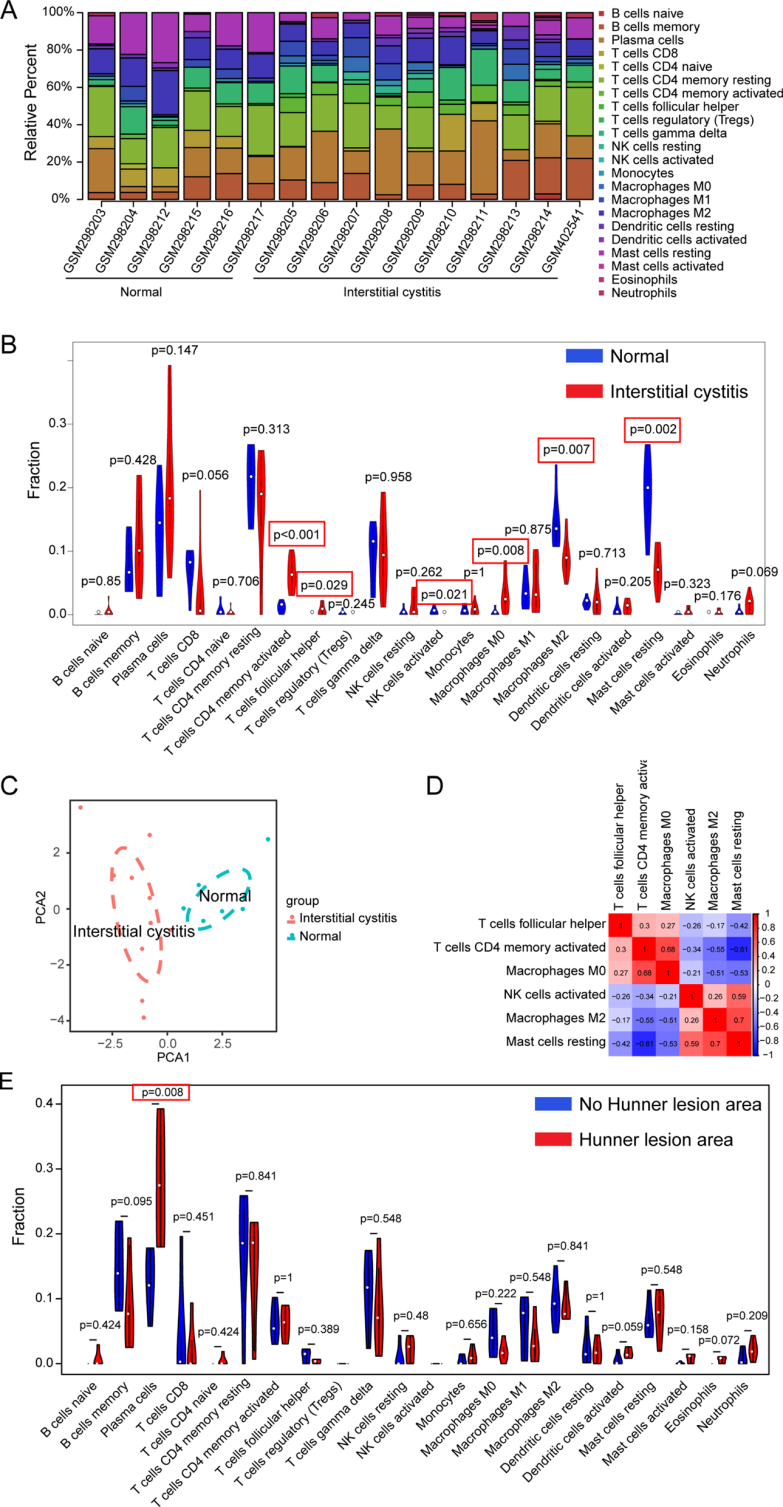
The landscape of immune infiltration in IC. **A** Bar charts of 22 immune cell proportions in HIC and normal tissues. **B** Differential expression of 22 immune cell subsets between HIC and normal tissues. **C** Principal component analysis of HIC and normal tissues. **D** Correlation matrix of six types of immune cell proportions. **E** Differential expression of 22 immune cell subsets between Hunner's lesion areas and non-Hunner's lesion areas

Figure 2A shows the proportions of immune cells in 16 bladder tissues. Memory B cells, CD4 + memory resting T cells, and plasma cells accounted for most infiltrating cells, especially in HIC tissue. The differential expressional proportion of immune infiltration cells in the HIC and control groups is shown in Fig. 2B. Six types of immune cells were differentially expressed: activated CD4+ memory T cells (*P* < 0.001); Tfh cells (*P* = 0.029); activated NK cells (*P* = 0.021); M0 macrophages (*P* = 0.008), M2 macrophages (*P* = 0.007); and resting mast cells (*P* = 0.002) (Fig. 2B). Activated CD4 memory T cells (*P* < 0.001), Tfh cells (*P* = 0.029) and M0 macrophages (*P* = 0.008) were upregulated in HIC tissue. Compared with healthy tissues, the increase in activated CD4 + memory T cells was the most significant in the HIC tissues. Resting mast cells and activated NK cells were downregulated significantly in HIC tissues (Fig. 2B). PCA showed that the proportions of immune cells from the tissues of HIC patients and healthy controls showed distinct group-bias clustering and individual differences (Fig. 2C).

Figure 2D displays the correlations between these significantly differentially expressed types of immune cells. The six types of immune cells were weakly to strongly correlated. Resting mast cells correlated positively with macrophages M2 (r = 0.7) and NK cells activated (0.59) and negatively with Tfh cells (r = –0.42), activated CD4^+^ memory T cells (r = –0.81), and M0 macrophages (r = –0.53). These results suggest that the function of resting mast cells activated CD4 + memory T cells, Tfh cells, and M0 macrophages in HIC may be antagonistic. However, the relationship between mast cells resting and activated NK cells was synergistic.

Unlike non-Hunner's lesion areas, in HIC samples, tissues of Hunner's lesion areas only expressed a higher proportion of plasma cells (Fig. 2E, *P* < 0.05).

### DEGs identification and GSEA assay

**Fig. 3:**
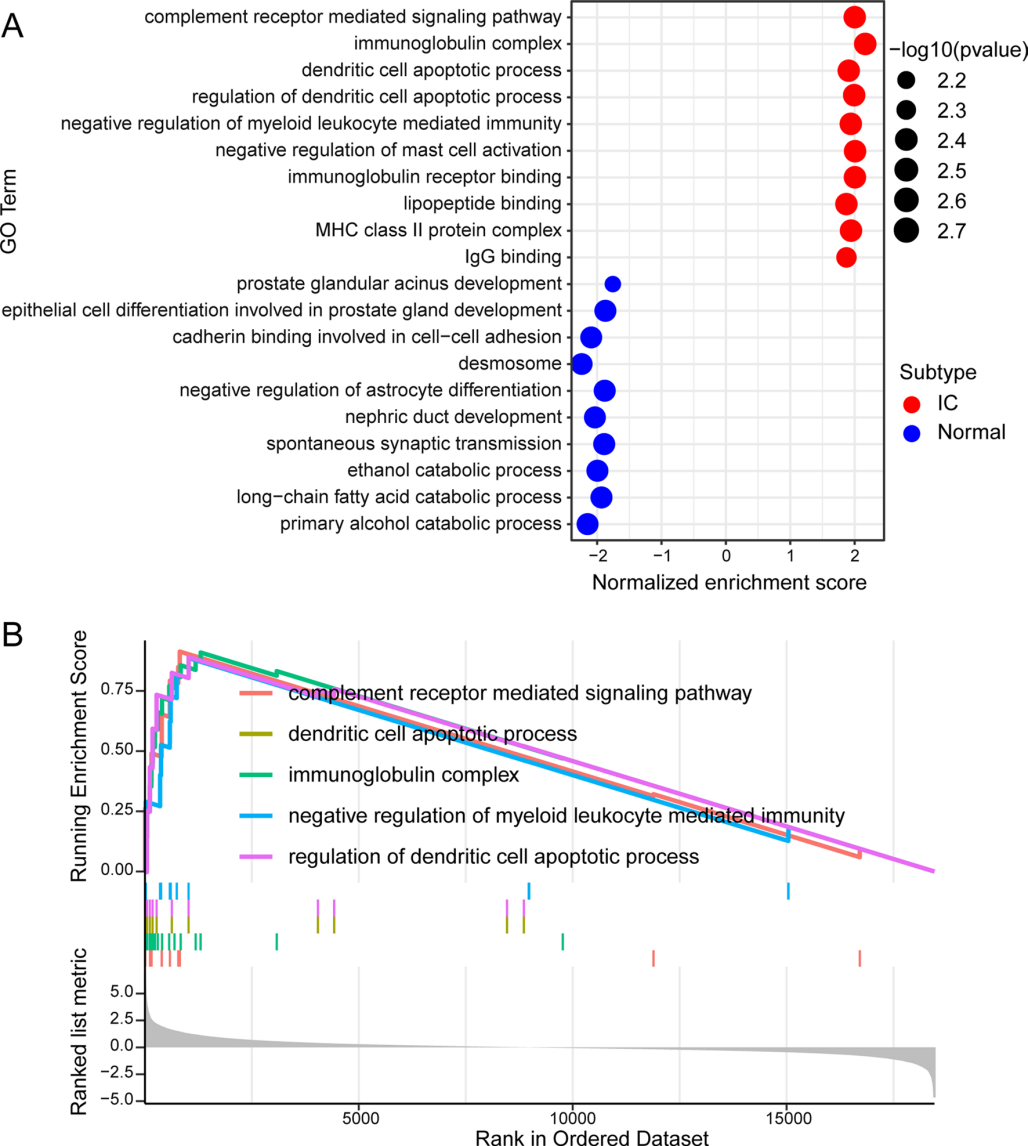
GO analysis results of GSEA. **A** Significantly enriched GO terms. The vertical objects are the names of GO terms, and the horizon axis represents the gene ratio. The depth of color represents the adjusted P-value. The area of a circle represents corresponding gene counts. **B** The top five activated GO terms of representative gene sets: complement receptor-mediated signaling pathway; dendritic cell apoptotic process; immunoglobulin complex; negative regulation of myeloid leukocyte mediated immunity; regulation of dendritic cell apoptotic process

A total of 4813 DEGs were identified between HIC and normal controls and are summarized in Additional file 1: Table S1. We then conducted the GSEA. Annotation of GO included molecular function, cellular components, and biological processes. GSEA generated the top ten most significantly enriched GO terms of each group (Fig. 3A). These enrichment results suggest that immune responses are crucial in HIC. The detailed GO results of GSEA are listed in Additional file 2: Table S2. The top five significantly activated GO terms are presented in Fig. 3B. This enrichment suggests that the activated gene sets were enriched in the front of the sequence.

**Fig. 4:**
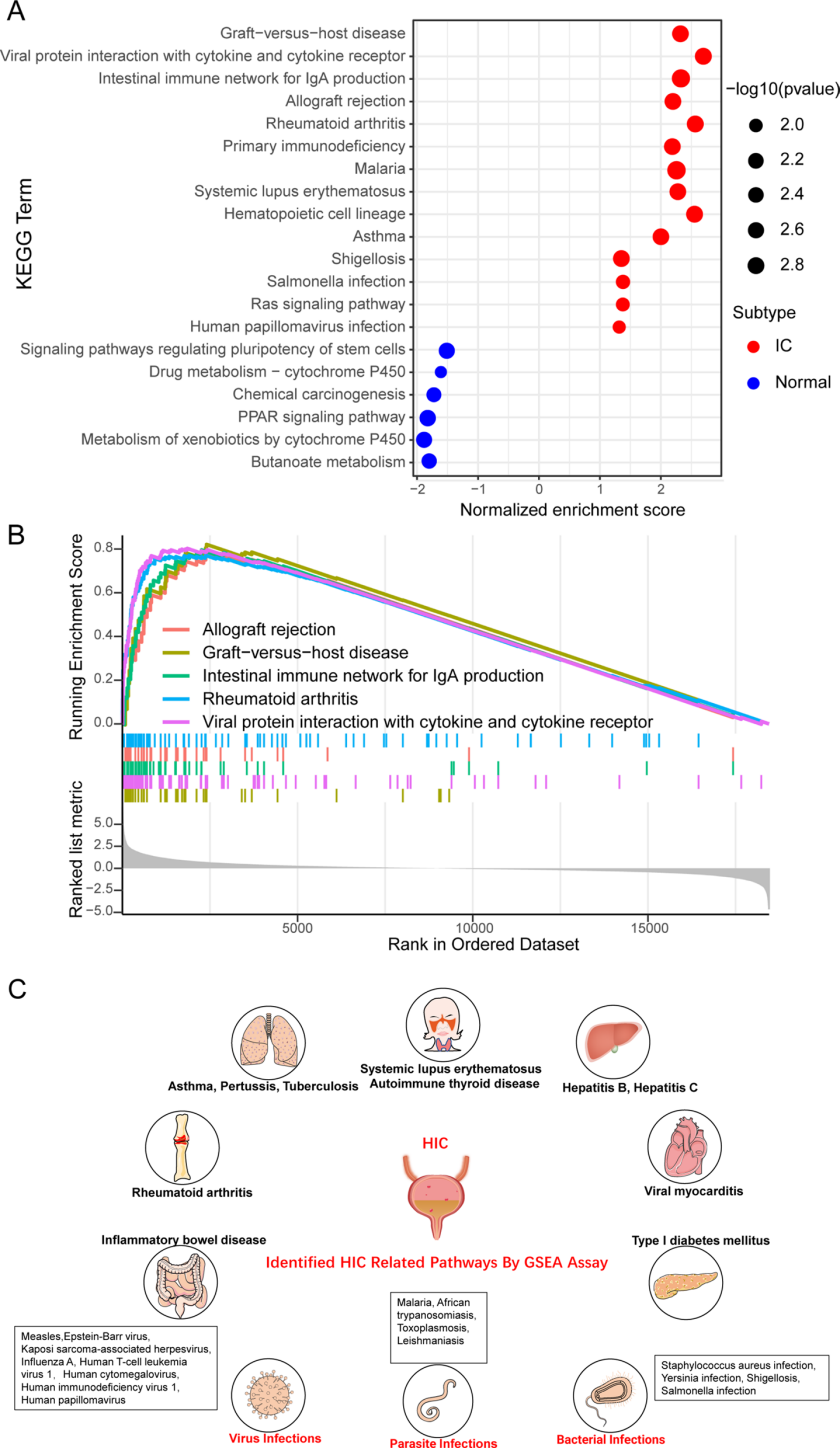
KEGG pathway analysis results of GSEA. **A** Significantly enriched KEGG pathways. The vertical objects are the names of KEGG pathway, and the horizon axis represents the gene ratio. The depth of color represents the adjusted p-values. The area of a circle represents corresponding gene counts. **B** GSEA-based KEGG-enrichment plots of representative gene sets from the top five activated pathway: Graft-versus-host disease; Viral protein interaction with cytokine and cytokine receptor; Intestinal immune network for IgA production; Allograft rejection; Rheumatoid arthritis. **C** Diagram of KEGG pathway results of GSEA. Autoimmune diseases and viral, parasitic, and bacterial infections related to HIC are summarized in the diagram

A total of 20 prominent KEGG pathways are shown in Fig. 4A. The top five significantly activated KEGG pathways are shown in Fig. 4B. The pathways related to autoimmune and infectious diseases are displayed in Table 1 and summarized in Fig. 4C. The detailed.

**Table 1:** 33 KEGG pathways of HIC related autoimmune and infectious diseases

No.	Description	Set size	Enrichment score	p value adjusted
1	Graft-versus-host disease	35	0.82	0.01
2	Intestinal immune network for IgA production	43	0.80	0.01
3	Rheumatoid arthritis	84	0.77	0.01
4	Malaria	46	0.77	0.01
5	Systemic lupus erythematosus	46	0.77	0.01
6	Asthma	26	0.76	0.01
7	Autoimmune thyroid disease	46	0.75	0.01
8	Type I diabetes mellitus	39	0.74	0.01
9	Leishmaniasis	71	0.73	0.01
10	Inflammatory bowel disease	61	0.70	0.01
11	Legionellosis	53	0.68	0.01
12	Pertussis	69	0.68	0.01
13	African trypanosomiasis	34	0.65	0.01
14	Tuberculosis	169	0.61	0.01
15	Viral myocarditis	54	0.61	0.01
16	Staphylococcus aureus infection	75	0.60	0.01
17	Measles	130	0.59	0.01
18	Epstein-Barr virus infection	191	0.59	0.01
19	Kaposi sarcoma-associated herpesvirus infection	183	0.58	0.01
20	Prion diseases	34	0.58	0.04
21	Toxoplasmosis	104	0.58	0.01
22	Influenza A	157	0.56	0.01
23	Human T-cell leukemia virus 1 infection	213	0.56	0.01
24	Hepatitis B	158	0.54	0.01
25	Human cytomegalovirus infection	214	0.51	0.01
26	Acute myeloid leukemia	66	0.50	0.02
27	Yersinia infection	117	0.46	0.01
28	Viral carcinogenesis	162	0.45	0.01
29	Human immunodeficiency virus 1 infection	196	0.44	0.01
30	Hepatitis C	149	0.39	0.05
31	Shigellosis	207	0.37	0.03
32	Salmonella infection	211	0.37	0.03
33	Human papillomavirus infection	317	0.33	0.04

KEGG pathways enrichment results of the GSEA assay are displayed in Additional file 3: Table S3. The results suggest that the activation of signaling pathways in HIC is similar to that of immune rejection diseases, infectious diseases, and autoimmune diseases such as rheumatoid arthritis and autoimmune thyroid disease.

### Expression analysis of 11 Tfh cell markers in GEO dataset GSE57560

Finally, the expression levels of 11 Tfh cell markers (CXCR5, PD-1, ICOS, SAP, CD200, IL-21, CXCR4, CXCL13, BTLA, SLAM, and CCR7) was analyzed in GSE57560 and compared between four IC patients with low capacity (< 400 ml) tissues and three normal bladder tissues. Then, presented as a scatter plot followed by statistical analysis using the Wilcoxon–Mann–Whitney test. Compared with the control group, the expression levels of 11 markers in all four patients were significantly increased.

## Discussion

Previous gene expression studies emphasized overexpression of pro‐inflammatory cytokines in IC [19, 20]. Recent evidence suggests that HIC is a distinct immune-related disease characterized by infiltration of several immune cells [10, 21, 22]. In recent years, new evidence revealed by RNAseq indicated that HIC is associated with significant upregulation of immune responses and infection-related biological processes [5]. However, few studies focused on the infiltration of multiple immune cell subsets, and immune cell biomarkers have yet been established.

This study uncovered differential expressional cell patterns of immune infiltration in HIC using the CIBERSORT algorithm. There was a significant difference in six immune cell subsets between HIC and healthy controls, with a rising trend in three subsets and a falling trend in another three (Figs. 1, 2B). Although the changes of these six immune cells are sufficient to distinguish HIC from healthy people using PCA (Fig. 2C), single-cell biomarkers are necessary for efficient diagnosis.

**Fig. 5:**
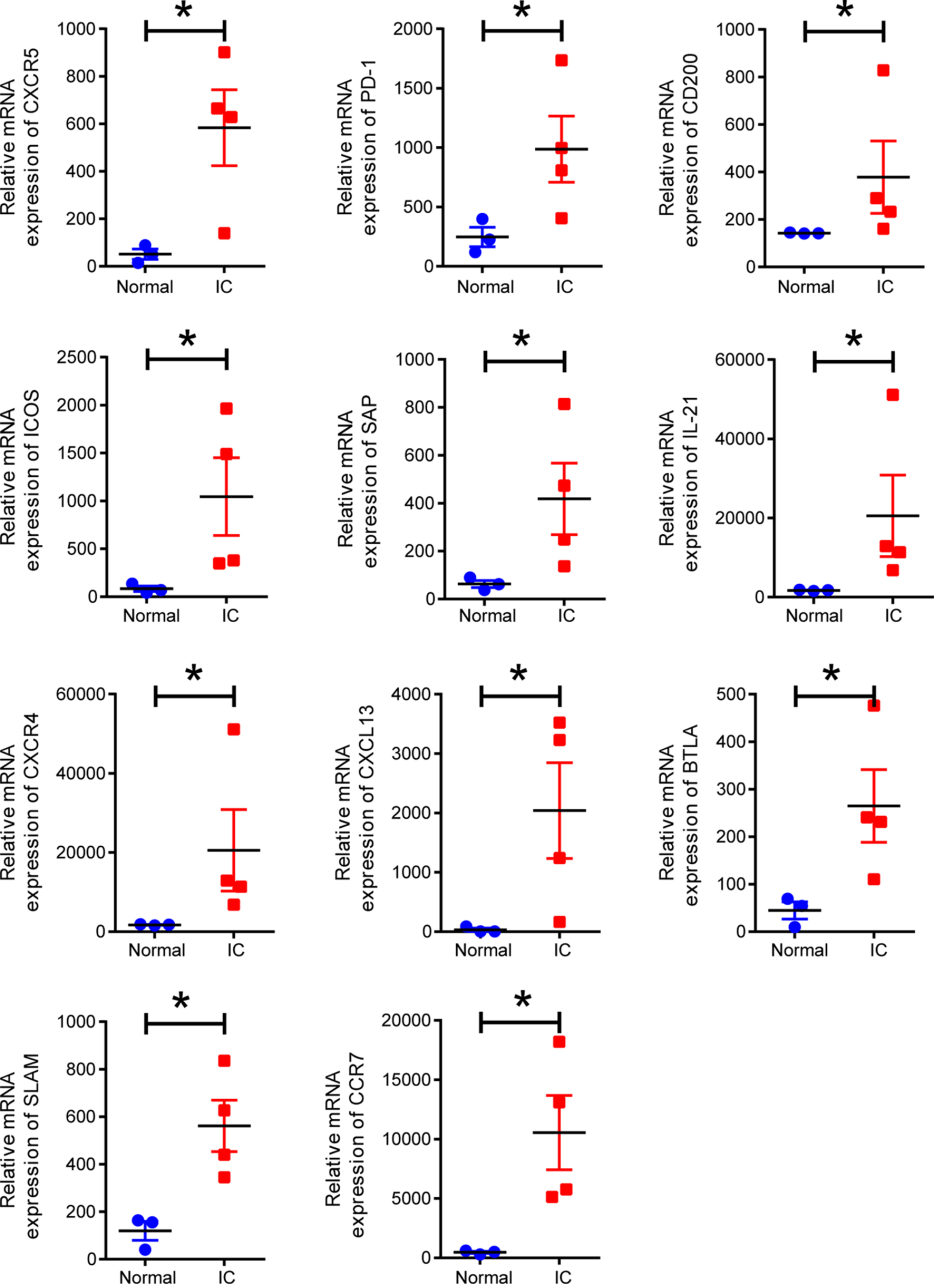
Expression of Tfh cell markers in IC/BPS datasets. Scatter plot data were acquired from GSE57560, which included comparing the expression levels of 11 Tfh cell markers between four IC/BPS patients with low capacity (< 400 ml) and three normal tissues. These scatter plots were made, and statistical analysis was conducted using GraphPad Prism. The *P*-value was calculated using the Wilcoxon–Mann–Whitney test. Star symbols indicate *P < 0.05

For the diagnosis of inflammatory diseases, elevated indicators are often more likely than falling indicators to be regarded as biomarkers. We noticed an exciting phenomenon in that the expression of Tfh cells was detected in HIC tissues (Fig. 2B); however, there was almost no expression in the normal controls. A study showed that Tfh cells facilitated autoimmune and B cell activation [23]. Overreaching Tfh cells can produce aberrant immune activation by excessive autoantibodies [24]. To further verify the effectiveness of Tfh cell as a biomarker, we tested 11 Tfh cell markers in additional samples from GSE57560, and all showed elevated expression in the patient group. Although we do not know which two of the four patients had HIC or NHIC, the expression of 11 markers in all four patients was increased (Fig. 5). These findings suggest that Tfh cells may serve as a HIC biomarker or even provide approaches for potential therapeutic strategies. We also predicted elevated expression of activated memory CD4^+^ T cells (CD3^+^ CD4^+^ HLA^−^DR^+^ cells) in HIC tissues (Fig. 2B). HLA-DR within T cells focuses on a subset of regulatory T cells, displaying regulatory and functional phenotypes [25]. In another study, a patient with polymyositis showed significantly elevated expression of CD3^+^ CD4^+^ HLA-DR^+^ cells in heparinized whole blood samples [26]. However, due to insufficient relevant literature, the potential roles of CD3^+^ CD4^+^ HLA-DR^+^ cells remain unclear. It remains uncertain whether HIC is a T cell-driven autoimmune disease with autoreactive T cells constantly tracked and activated by self MHC II peptides. If this possibility exists, immunosuppressive biologics targeting autoreactive CD4^+^ T cells may offer an opportunity to treat HIC/IC. Memory CD4^+^ T cells can archive information about their activation, helping them elicit rapid effector responses such as interferon-gamma secretion upon reactivation [27]. However, the rapid secretion of high levels of chemokines or cytokines can be harmful [27].

We also found increased expression of M0 macrophages and decreased expression of M2 macrophages (Fig. 2B). Unstimulated macrophages or those in resting status are referred to as M0 subsets [28]. M0 macrophages (the resting phenotype) can be polarized into subsets of M1 (pro-inflammatory) or M2 (anti-inflammatory) [28]. M2 macrophages are associated with immunosuppression and anti-inflammatory effects and promote tissue remodeling, healing, and repairing [29, 30]. These anti-inflammatory phenotypes can be induced from undifferentiated M0 macrophages by cytokines [31]. These findings suggest that the transformation of macrophage subsets and damaging the process of anti-inflammation by M2 macrophages may explain the etiology of HIC. Modulation of macrophage reprogramming and polarization in HIC may be a promising treatment option for HIC patients.

NK cells showed decreasing HIC trends (Fig. 2B), which can be explained as an exhausted status like chronic infections or tumors [32]. Given the shorter lifespan of NK cells, they are poor candidates for diagnostic biomarkers. However, the cellular and molecular mechanisms of the exhausted status of NK cells in HIC remain a question worth exploring.

Among the types of immune cells showing altered expression, resting mast cells showed a decreasing trend, accompanied by an increasing trend of activated mast cells (Fig. 2B). Mast cells are turned off in the resting state and turned on to degranulate when activated [33]. Under resting states, the mast cells use a plastic repertoire to limit immune functions in the absence of microenvironment activation [33]. Reports of mast cell density in HIC/IC appear to be controversial [4]. Our findings suggest that decreased expression of resting mast cells may participate in the loss of balance between immunity and tolerance to inflammatory processes in HIC.

Chronic inflammation is not confined to the Hunner lesion; it is also found in areas outside the area of the Hunner lesions; these are features of “pancystitis” [4]. Nevertheless, there remain differences in the expression of immune cells in these two regions. In the present study, there were more plasma cells in Hunner lesion area tissues than non-Hunner lesion area tissues (Fig. 2E), consistent with a previous study [34]. Plasma cells can maintain the balance of autoimmunity and inflammatory processes [35]. However, long-lived plasma cells contribute to chronic inflammation in autoimmune diseases by continuous secretion of pathogenic antibodies, causing symptom flares [35]. For these reasons, targeted depletion of these plasma cells might serve as a potentially curative therapy.

Most GO terms in the GSEA assay were immune-related terms like “negative regulation of mast cell activation” and “dendritic cell apoptotic process” (Fig. 3A, B). This finding suggests the immunological properties and pathogenesis of HIC. Autoimmune diseases such graft-versus-host disease, rheumatoid arthritis, and systemic lupus erythematosus were highly associated with HIC pathways according to the GSEA (Fig. 4A, C). A recent population-based cohort study identified a concordance of IC with autoimmune diseases like systemic lupus erythematosus [36]. The immunoreaction of HIC and responses to these immune diseases may share several common biology processes or even similar therapy strategies.

We also found that infectious diseases such as malaria and human papillomavirus infection may be associated with HIC (Fig. 4A, C). A recent study found evidence of two human polyomavirus infections in IC urine samples; this finding hints at a viral etiology [37]. Therefore, in the diagnosis of HIC, careful screening for autoimmune diseases and microbial infection-related indicators might be valuable to determine the etiology.

The present study is the first to characterize immune cell infiltration in HIC. All gene expression data were downloaded from the GEO database and were therefore reliable. The results of CIBERSORT and GSEA assays were mutually supportive, suggesting insights for novel biomarker development. Whether Tfh cells and CD3^+^ CD4^+^ HLA-DR^+^ cells as diagnostic biomarkers can be extended from HIC patients to all IC patients requires further verification. Although this study used two data sets from GEO to cross-validate the results of CIBERSORT analysis, the lack of verification of more biological samples is a limitation of this study. The scarcity of data available for IC/HIC is another limitation. Nevertheless, we were fortunate to obtain many novel and significant results.

## Conclusions

The current study is the first to characterize the infiltration of several immune cell subsets in HIC samples. Tfh cells and CD3^+^ CD4^+^ HLA-DR^+^ cells may serve as novel diagnostic biomarkers for HIC disease.

## Supplementary Information

**Additional file 1**: DEGs between HIC and healthy controls.

**Additional file 2**: The detailed GO results of GSEA assay.

**Additional file 3**: The detailed KEGG results of GSEA assay.

## Data Availability

All the data analyzed in this study are included in NCBI GEO database GSE11783, GSE 57560, https://www.ncbi.nlm.nih.gov/geo/query/acc.cgi?acc=GSE11783, https://www.ncbi.nlm.nih.gov/geo/query/acc.cgi?acc=GSE57560.

## Abbreviations

BPS: Bladder Pain Syndrome
IC: Interstitial Cystitis
HIC: Interstitial Cystitis with Hunner's lesion
NHIC: Non-Hunner lesion Interstitial Cystitis
GSEA: Gene Set Enrichment Analysis
Tfh cells: T cells follicular helper cells
DEGs: Differentially expressed genes
PCA: Principal Component Analysis

## References

[1] Peeker R, Fall M (2000). Treatment guidelines for classic and non-ulcer interstitial cystitis. Int Urogynecol J Pelvic Floor Dysfunct.

[2] Peters KM, Killinger KA, Mounayer MH, Boura JA (2011). Are ulcerative and nonulcerative interstitial cystitis/painful bladder syndrome 2 distinct diseases? A study of coexisting conditions. Urology.

[3] Fall M, Nordling J, Cervigni M, Dinis Oliveira P, Fariello J, Hanno P (2020). Hunner lesion disease differs in diagnosis, treatment and outcome from bladder pain syndrome: an ESSIC working group report. Scand J Urol.

[4] Whitmore KE, Fall M, Sengiku A, Tomoe H, Logadottir Y, Kim YH (2019). Hunner lesion versus non-Hunner lesion interstitial cystitis/bladder pain syndrome. Int J Urol.

[5] Akiyama Y, Maeda D, Katoh H, Morikawa T, Niimi A, Nomiya A (2019). Molecular taxonomy of interstitial cystitis/bladder pain syndrome based on whole transcriptome profiling by next-generation RNA sequencing of bladder mucosal biopsies. J Urol.

[6] Gamper M, Viereck V, Geissbuhler V, Eberhard J, Binder J, Moll C (2009). Gene expression profile of bladder tissue of patients with ulcerative interstitial cystitis. BMC Genom.

[7] Tseng LH, Chen I, Wang CN, Lin YH, Lloyd LK, Lee CL (2010). Genome-based expression profiling study of Hunner's ulcer type interstitial cystitis: an array of 40-gene model. Int Urogynecol J.

[8] Theoharides TC, Sant GR, El-Mansoury M, Letourneau R, Ucci AA, Meares EM (1995). Activation of bladder mast cells in interstitial cystitis: a light and electron microscopic study. J Urol.

[9] Maeda D, Akiyama Y, Morikawa T, Kunita A, Ota Y, Katoh H (2015). Hunner-type (classic) interstitial cystitis: a distinct inflammatory disorder characterized by pancystitis, with frequent expansion of clonal B-cells and epithelial denudation. PLoS ONE.

[10] Akiyama Y, Yao JR, Kreder KJ, O'Donnell MA, Lutgendorf SK, Lyu D (2021). Autoimmunity to urothelial antigen causes bladder inflammation, pelvic pain, and voiding dysfunction: a novel animal model for Hunner-type interstitial cystitis. Am J Physiol Renal Physiol.

[11] Keay S, Zhang CO, Trifillis AL, Hebel JR, Jacobs SC, Warren JW (1997). Urine autoantibodies in interstitial cystitis. J Urol.

[12] Shen-Orr SS, Gaujoux R (2013). Computational deconvolution: extracting cell type-specific information from heterogeneous samples. Curr Opin Immunol.

[13] Newman AM, Liu CL, Green MR, Gentles AJ, Feng W, Xu Y (2015). Robust enumeration of cell subsets from tissue expression profiles. Nat Methods.

[14] Reimand J, Isserlin R, Voisin V, Kucera M, Tannus-Lopes C, Rostamianfar A (2019). Pathway enrichment analysis and visualization of omics data using g:Profiler, GSEA, Cytoscape and EnrichmentMap. Nat Protoc.

[15] Kanehisa M (2019). Toward understanding the origin and evolution of cellular organisms. Prot Sci.

[16] Kanehisa M, Furumichi M, Sato Y, Ishiguro-Watanabe M, Tanabe M (2021). KEGG: integrating viruses and cellular organisms. Nucleic Acids Res.

[17] Kanehisa M, Goto S (2000). KEGG: kyoto encyclopedia of genes and genomes. Nucleic Acids Res.

[18] Crotty S (2011). Follicular helper CD4 T cells (TFH). Annu Rev Immunol.

[19] Homma Y, Nomiya A, Tagaya M, Oyama T, Takagaki K, Nishimatsu H (2013). Increased mRNA expression of genes involved in pronociceptive inflammatory reactions in bladder tissue of interstitial cystitis. J Urol.

[20] Ogawa T, Homma T, Igawa Y, Seki S, Ishizuka O, Imamura T (2010). CXCR3 binding chemokine and TNFSF14 over expression in bladder urothelium of patients with ulcerative interstitial cystitis. J Urol.

[21] Fall M, Johansson SL, Vahlne A (1985). A clinicopathological and virological study of interstitial cystitis. J Urol.

[22] Wang X, Liu W, O'Donnell M, Lutgendorf S, Bradley C, Schrepf A (2016). Evidence for the role of mast cells in cystitis-associated lower urinary tract dysfunction: a multidisciplinary approach to the study of chronic pelvic pain research network animal model study. PLoS ONE.

[23] Vinuesa CG, Linterman MA, Yu D, MacLennan IC (2016). Follicular helper T cells. Annu Rev Immunol.

[24] Scherm MG, Ott VB, Daniel C (2016). Follicular helper T cells in autoimmunity. Curr Diab Rep.

[25] Revenfeld ALS, Baek R, Jorgensen MM, Varming K, Stensballe A (2017). Induction of a regulatory phenotype in CD3+ CD4+ HLA-DR+ T cells after allogeneic mixed lymphocyte culture; indications of both contact-dependent and -independent activation. Int J Mol Sci.

[26] Ishii W, Matsuda M, Shimojima Y, Itoh S, Sumida T, Ikeda S (2008). Flow cytometric analysis of lymphocyte subpopulations and TH1/TH2 balance in patients with polymyositis and dermatomyositis. Intern Med.

[27] Jaigirdar SA, MacLeod MK (2015). Development and function of protective and pathologic memory CD4 T cells. Front Immunol.

[28] Miao X, Leng X, Zhang Q (2017). The current state of nanoparticle-induced macrophage polarization and reprogramming research. Int J Mol Sci.

[29] Lawrence T, Natoli G (2011). Transcriptional regulation of macrophage polarization: enabling diversity with identity. Nat Rev Immunol.

[30] Martinez FO, Helming L, Gordon S (2009). Alternative activation of macrophages: an immunologic functional perspective. Annu Rev Immunol.

[31] Stein M, Keshav S, Harris N, Gordon S (1992). Interleukin 4 potently enhances murine macrophage mannose receptor activity: a marker of alternative immunologic macrophage activation. J Exp Med.

[32] Campbell KS, Hasegawa J (2013). Natural killer cell biology: an update and future directions. J Allergy Clin Immunol.

[33] Frossi B, Mion F, Tripodo C, Colombo MP, Pucillo CE (2017). Rheostatic functions of mast cells in the control of innate and adaptive immune responses. Trends Immunol.

[34] Akiyama Y, Morikawa T, Maeda D, Shintani Y, Niimi A, Nomiya A (2016). Increased CXCR3 expression of infiltrating plasma cells in Hunner type interstitial cystitis. Sci Rep.

[35] Hiepe F, Dorner T, Hauser AE, Hoyer BF, Mei H, Radbruch A (2011). Long-lived autoreactive plasma cells drive persistent autoimmune inflammation. Nat Rev Rheumatol.

[36] Wen JY, Lo TS, Chuang YC, Ho CH, Long CY, Law KS (2019). Risks of interstitial cystitis among patients with systemic lupus erythematosus: a population-based cohort study. Int J Urol.

[37] Robles MTS, Cantalupo PG, Duray AM, Freeland M, Murkowski M, van Bokhoven A (2020). Analysis of viruses present in urine from patients with interstitial cystitis. Virus Genes.

